# Large Cell Neuroendocrine Carcinoma With Acquired Hemophilia A Diagnosed by Endobronchial Biopsy: A Case Report

**DOI:** 10.7759/cureus.70623

**Published:** 2024-10-01

**Authors:** Kei Yaoita, Toshikazu Takasaki, Shoko Ito, Toshimi Kawahata, Makoto Maemondo

**Affiliations:** 1 Division of Pulmonary Medicine, Department of Medicine, Jichi Medical University, Tochigi, JPN; 2 Division of Hematology, Department of Medicine, Jichi Medical University, Tochigi, JPN

**Keywords:** acquired hemophilia a, bronchoscopy, endobronchial biopsy, large cell neuroendocrine carcinoma, lung cancer

## Abstract

Primary lung cancer with acquired hemophilia A is rare. We report a case of large cell neuroendocrine carcinoma (LCNEC) with acquired hemophilia A diagnosed by endobronchial biopsy. A 75-year-old male was admitted to our hospital due to severe anemia and multiple organ failure. Hematomas, prolonged activated partial thromboplastin time, decreased activity of factor VIII, and the presence of serum factor VIII inhibitors led to the diagnosis of acquired hemophilia A. Administration of recombinant activated factor VIII and steroid treatment resolved the coagulation abnormalities. Simultaneously, a CT scan showed a lung mass and an endobronchial biopsy confirmed the diagnosis of LCNEC. Although acquired hemophilia A can cause severe bleeding symptoms, a lung cancer diagnosis by bronchoscopy was possible under appropriate treatment of acquired hemophilia A.

## Introduction

Acquired hemophilia A is a rare disease characterized by the production of autoantibodies against factor VIII, resulting in decreased activity of factor VIII and bleeding symptoms. It has been reported that acquired hemophilia A is associated with autoimmune diseases, malignancies, pregnancy, and others [1]. Cases of acquired hemophilia A with primary lung cancer have rarely been reported. In patients with acquired hemophilia A who have underlying malignant tumors, bleeding tends to be severe, and early diagnosis along with effective hemostatic treatment significantly impacts prognosis [2]. In this report, we present a specific case of large cell neuroendocrine carcinoma (LCNEC) co-occurring with acquired hemophilia A, which was diagnosed by endobronchial biopsy.

## Case presentation

The patient was a 75-year-old man with a 90-pack-year smoking history. He had comorbidities of diabetes mellitus, hypertension, and dyslipidemia. Three months prior to the clinic visit, he experienced subcutaneous hematomas in both lower limbs, which spontaneously resolved. After that, as a subcutaneous hematoma on the right forearm with a general sense of malaise appeared, he visited a clinic. Severe anemia and multiple organ failure were revealed there, and he was admitted to our hospital.

**Figure 1:**
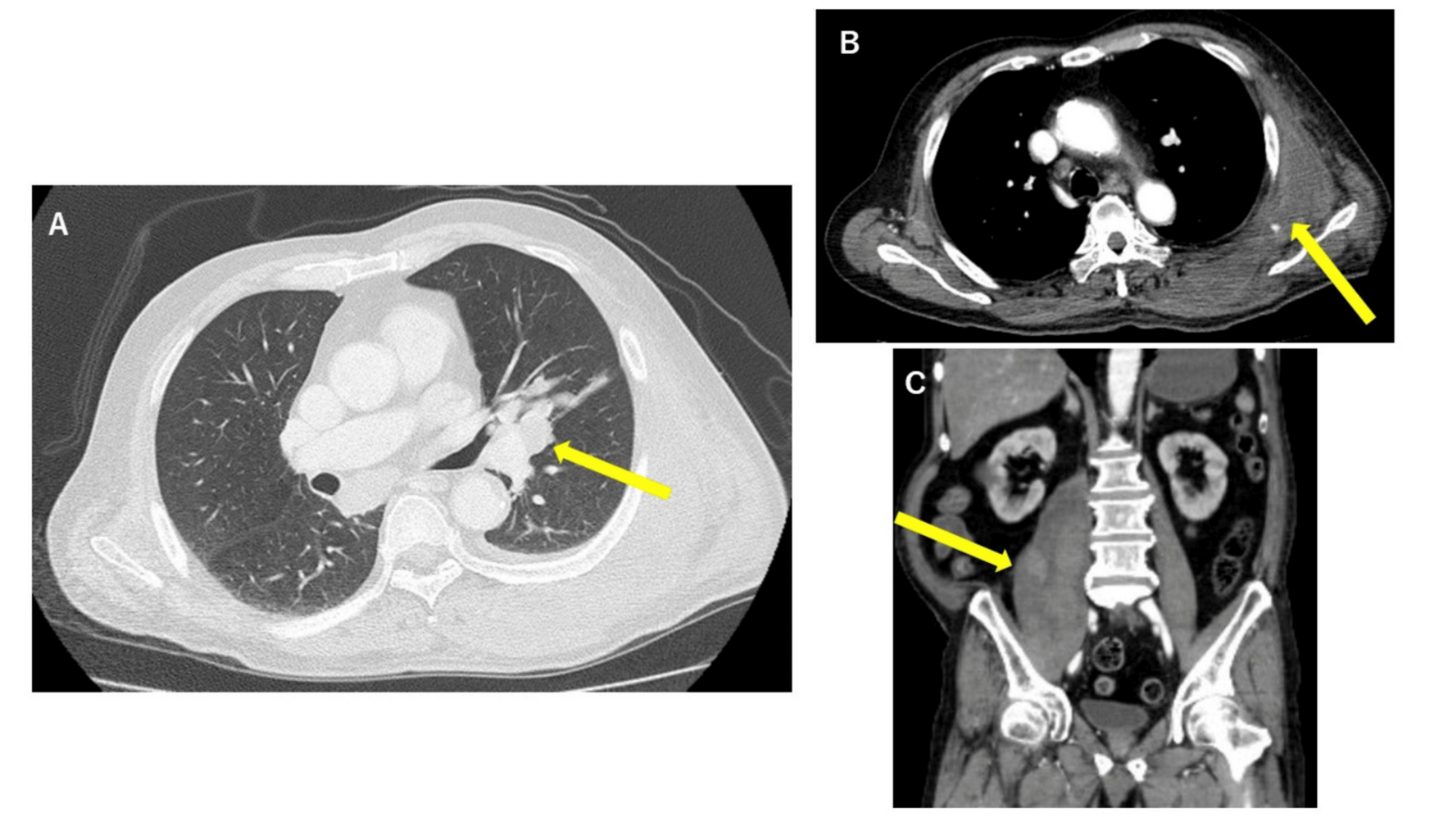
Contrast-enhanced CT of the thorax and abdomen A: Axial view, showing a 45 mm mass in the left pulmonary hilar region. B: Axial view, showing hematomas in the left back intramuscularly and subcutaneously. C: Coronal view, showing a hematoma in the right iliopsoas muscle.

On admission, physical information and vital signs were as follows: height 160 cm, weight 71 kg, body temperature 35.1°C, pulse 92/min, blood pressure 129/70 mmHg, respiratory rate 32/min, and SpO_2_ 96% (in room air). Physical examination revealed a regular heart sound with no murmur, no pulmonary rales, and subcutaneous hematomas were observed on the right forearm, left side of the abdomen to the back, and posterior aspect of the left thigh. Laboratory data are shown in Table 1. Hemoglobin (Hb) level was 4.7 g/dL and platelets counted at 57.2 × 10^4^/μL. Severe anemia and thrombocytopenia were observed. The prothrombin time-international normalized ratio registered at 1.30 and activated partial thromboplastin time (APTT) was prolonged at 70.4 seconds, indicative of coagulopathy. Biochemistry results revealed elevated creatinine and liver dysfunction with elevated bilirubin levels. Tumor markers of cytokeratin 19 fragment and neuron-specific enolase were elevated. Arterial blood gas analysis revealed lactic acidosis with pH 7.22 and lactate 18.0 mmol/L. CT scans showed a 45 mm mass in the left pulmonary hilar region, along with enlarged mediastinal lymph nodes (Figure 1A). Multiple small nodules were observed in the right lung. Hematomas were also observed in the right iliopsoas muscle and the left back, both intramuscularly and subcutaneously (Figure 1B, 1C).

**Table 1:** Laboratory data on admission

Parameter	Result	Reference range
Hematological test		
White blood cells (/μL)	38,000	3,300-8,600
Neutrophils (%)	89.4	40.0-71.0
Eosinophils (%)	0.1	0.2-6.8
Lymphocytes (%)	5.4	26.2-46.6
Basophils (%)	0.5	0.0-1.0
Monocytes (%)	4.6	2.3-7.7
Red blood cells (×10^4^/μL)	152	435-555
Hemoglobin (g/dL)	4.7	13.7-16.8
Hematocrit (%)	15.5	40.7-50.1
Mean corpuscular volume (fL)	101.9	83.6-98.2
Platelet (×10^4^/μL)	57.2	15.8-34.8
Prothrombin time-international normalized ratio	1.3	0.85-1.15
Activated partial thromboplastin time (s)	70.3	22.4-37.4
D-dimer (μg/mL)	13.9	＜1
Fibrinogen (mg/dL)	424	200-400
von Willebrand factor (%)	334	50-150
Factor Ⅻ activity (%)	29	46-156
Factor Ⅺ activity (%)	84	73-136
Factor Ⅸ activity (%)	68.9	60-140
Factor Ⅷ activity (%)	3.4	60-140
FⅧ inhibitor (BU/mL)	9.2	Negative
Biochemical test		
Albumin (g/dL)	3.1	4.1-5.1
Blood urea nitrogen (mg/dL)	57	8.0-20
Creatinine (mg/dL)	2.93	0.65-1.07
Total bilirubin (mg/dL)	2.12	0.4-1.5
Aspartate aminotransferase (U/L)	4,272	13-30
Alanine aminotransferase (U/L)	2,575	10-42
Lactate dehydrogenase (U/L)	8,768	124-222
Creatine phosphokinase (U/L)	431	59-248
Sodium (mmol/L)	138	138-145
Potassium (mmol/L)	6.1	3.6-4.8
Chloride (mmol/L)	98	101-108
Calcium (mg/dL)	8.9	8.8-10.1
Tumor marker		
Carcinoembryonic antigen (ng/mL)	4.9	<4.5
Cytokeratin 19 fragment (ng/mL)	21	<3.5
Neuron-specific enolase (ng/mL)	57.3	<12.0
Progastrin-releasing peptide (pg/mL)	52	<81
Arterial blood gas analysis		
pH	7.22	7.35-7.45
Partial pressure of carbon dioxide (Torr)	15.7	35-45
Partial pressure of oxygen (Torr)	125	80-100
Bicarbonate (mmol/L)	6.3	20-26
Anion gap (mmol/L)	33.7	12-16
Lactate (mmol/L)	18	0.5-2.0

**Figure 2:**
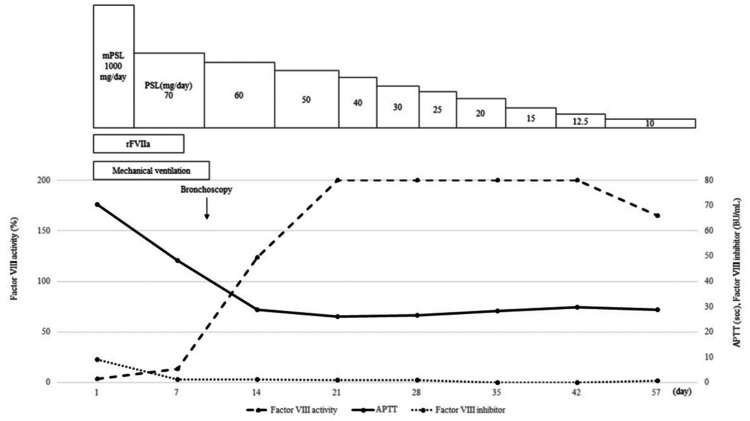
Clinical course of the patient mPSL, methylprednisolone; PSL, prednisolone; rFⅦa, recombinant activated factor VII; APTT, activated partial thromboplastin time; BU, Bethesda Units.

**Figure 3:**
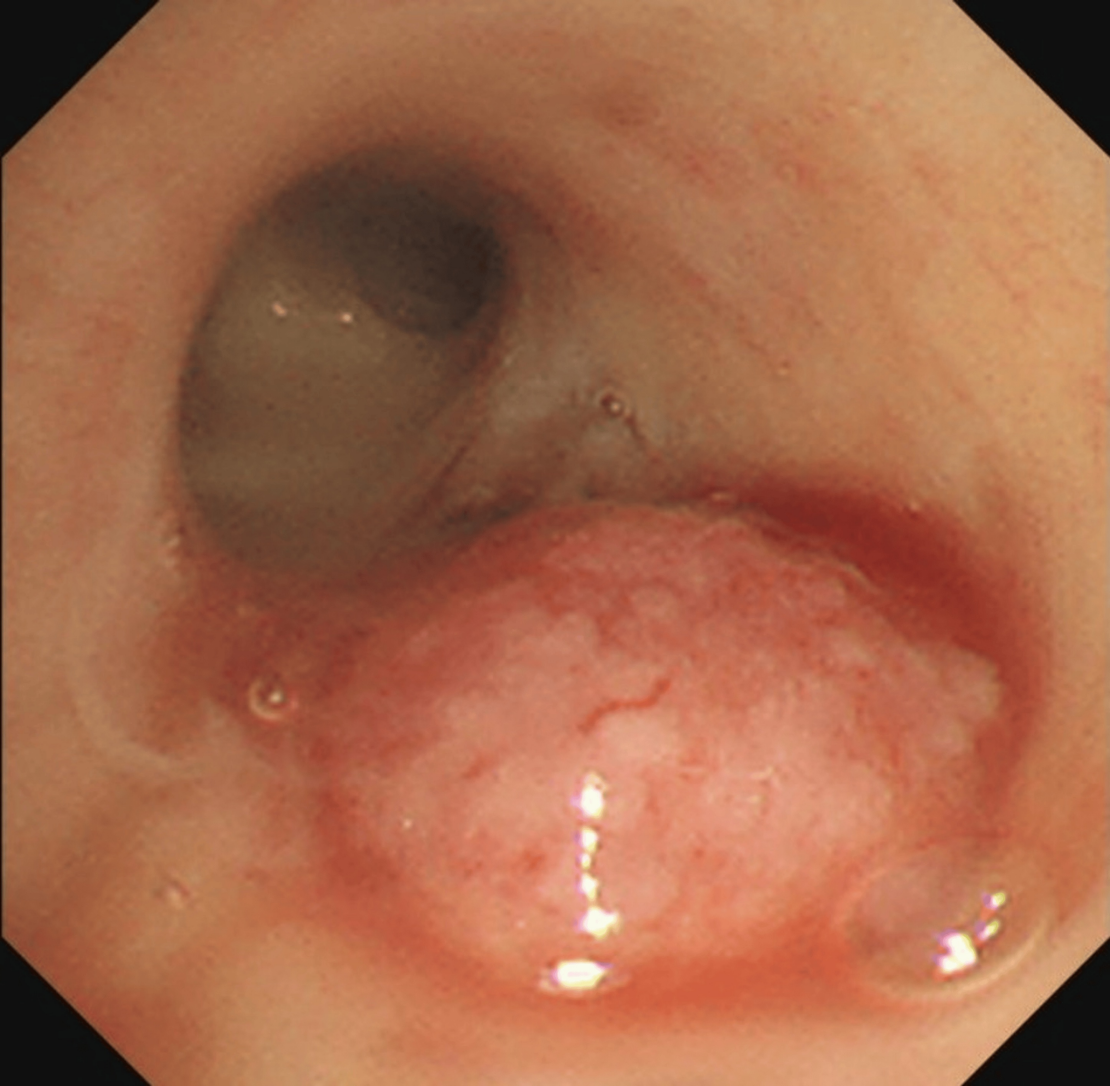
A bronchoscopic image The image shows a protruding lesion with granular surface changes at the entrance of the left upper lobe.

**Figure 4:**
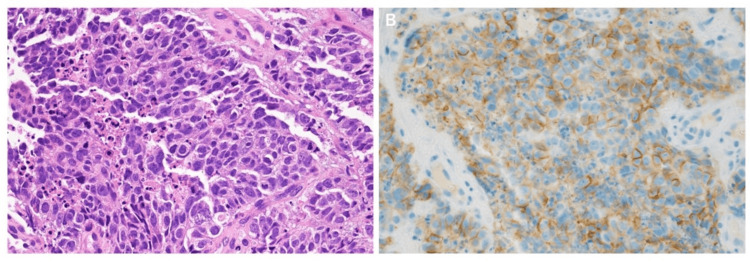
The histopathological findings and immunohistochemical staining A: Hematoxylin-eosin (HE) staining. B: CD56.

The patient was admitted to the intensive care unit due to multiple organ failure. Although there were no airway issues, respiratory compensation for metabolic acidosis was insufficient, raising concerns about respiratory collapse. As a result, ventilatory management was initiated. Given the presence of subcutaneous and intramuscular hemorrhage and the prolonged APTT, acquired hemophilia was suspected. Decreased factor VIII activity (3.4%, reference range: 60-140%) and positive factor VIII inhibitors (9.2 Bethesda Units/mL) were detected, and he was diagnosed with acquired hemophilia A. Recombinant activated factor VII and high-dose steroid treatment was initiated to improve severe bleeding symptoms. Steroids were administered, starting with methylprednisolone at 1000 mg/day for three days, followed by prednisolone (PSL) at 70 mg/day, which was gradually tapered (Figure 2). After initiation of treatment, the coagulation abnormalities improved. On the ninth day of hospitalization, a bronchoscopy was performed under mechanical ventilation to examine the pulmonary mass. The examination with bronchoscopy revealed a gently elevated lesion with granular surface changes at the entrance of the left upper lobe bronchus (Figure 3), and an endobronchial biopsy was performed with minimal bleeding. Pathological findings showed carcinoma cells characterized by densely stained enlarged nuclei with prominent nuclear division and necrosis (Figure 4). Immunostaining was negative for TTF-1 and p40, weakly positive for chromogranin A, and positive for CD56, leading to an LCNEC diagnosis. Brain MRI and bone scintigraphy did not indicate metastasis, and the clinical stage was diagnosed as T2aN3M1a, stage IVA.

The patient was extubated and weaned from the ventilator on the ninth day of mechanical ventilation. Acquired hemophilia A was well controlled, and there was no significant deterioration in LCNEC disease. However, disuse progressed, and performance status declined. We considered that anticancer chemotherapy was inappropriate for this patient with a compromised performance status of 3 and cognitive dysfunction and selected the approach of the best supportive care. The acquired hemophilia A responded well to the treatment with steroids and administration of factor VII, with factor VIII inhibitor levels measured on the eighth day after initiation of treatment decreasing to 1.2 Bethesda Units/mL and not rising again. Factor VIII activity also remained normalized. PSL was tapered to 10 mg/day, and the patient was transferred to a rehabilitation hospital. Although the tumor showed a tendency to enlarge, no life-threatening lesions appeared, and the respiratory condition remained stable until the transfer.

## Discussion

We encountered a case of LCNEC accompanied by acquired hemophilia A, diagnosed by bronchoscopy with endobronchial biopsy. Acquired hemophilia A is a rare disease, with an approximate incidence of one per million per year. Malignant tumors are identified in 10-15% of patients with acquired hemophilia A. Among solid tumors, lung and prostate cancers are common in the global data [2,3]. A systematic review by Napolitano et al. reported that lung cancer accounted for 15.8% of solid tumors [2]. On the other hand, in Japan, gastric and colorectal cancers are more common [4]. Although the precise mechanism of the production of factor VIII inhibitors in cancer patients remains unclear, it may be attributed to disturbances in immune tolerance, driven by factors such as aging, tumor characteristics, environmental elements, and genetic factors [5]. Previous reports of primary lung cancer associated with acquired hemophilia A have shown both early and advanced stages, with various histological types [5-18]. However, this is the first reported case of LCNEC combined with acquired hemophilia A.

In this case, LCNEC was diagnosed by bronchoscopy with endobronchial biopsy. The patient experienced severe bleeding symptoms during the initial assessment. However, following the administration of recombinant activated factor VII and systemic steroids to address the coagulopathy, the endobronchial biopsy was performed safely. Acquired hemophilia A is characterized by severe bleeding symptoms, including extensive subcutaneous and intramuscular hemorrhages [19]. Achieving hemostasis during the treatment of the initial symptoms is not easy. Treatment primarily comprises hemostatic measures and immunosuppressive therapy. Hemostatic treatment includes the use of bypass hemostatic agents, such as recombinant activated factor VII and activated prothrombin complex concentrate. Immunosuppressive therapy includes PSL alone or in combination with cyclophosphamide. In this case, the administration of recombinant activated factor VII and systemic steroids worked effectively.

A prior report on acquired hemophilia A associated with malignancy indicated that complete coagulation remission was attained in approximately 60-70% of patients [2,3]. There is also evidence that the inhibitor vanished after malignancy treatment alone, suggesting that acquired hemophilia A might be considered a tumor-associated syndrome [3]. In cases of primary lung cancer, coagulopathic remission has been reported following surgery or anticancer chemotherapy alone [17,18]. In this case, since the patient had not received treatment for LCNEC and the tumor was not under control, it is difficult to determine from the clinical course whether it was a tumor-associated syndrome. However, treatment with recombinant activated factor VII and steroids alone, without chemotherapy, was effective for acquired hemophilia A. Primary lung cancer concurrent with acquired hemophilia A is rare and often presents with severe symptoms. However, appropriate treatment can improve prognosis, and a bronchoscopic biopsy can be safely performed once coagulation abnormalities are controlled.

## Conclusions

We report a case of LCNEC with acquired hemophilia A diagnosed by endobronchial biopsy. The patient presented with severe bleeding symptoms at the time of initial diagnosis, but treatment with recombinant activated factor VII and steroids improved coagulation abnormalities, which led to the diagnosis of lung cancer. Due to poor performance status, the patient could not receive treatment for lung cancer. Although the tumor showed a tendency to enlarge, acquired hemophilia A remained well controlled. Primary lung cancer accompanied by acquired hemophilia A is rare, but appropriate initial management can improve the prognosis. Therefore, it is important to consider this condition in the differential diagnosis for lung cancer patients with bleeding symptoms.
